# Oral Amoxicillin Versus Benzyl Penicillin for Severe Pneumonia Among Kenyan Children: A Pragmatic Randomized Controlled Noninferiority Trial

**DOI:** 10.1093/cid/ciu1166

**Published:** 2014-12-30

**Authors:** Ambrose Agweyu, David Gathara, Jacquie Oliwa, Naomi Muinga, Tansy Edwards, Elizabeth Allen, Elizabeth Maleche-Obimbo, Mike English, for the Severe Pneumonia Study Group, Florence Aweyo, Bernard Awuonda, Martin Chabi, Newton Isika, Mary Kariuki, Magdalene Kuria, Polycarp Mandi, Leah Masibo, Thaddeus Massawa, Wycliffe Mogoa, Beatrice Mutai, Gatwiri Muriithi, Samuel Ng'arng'ar, Rachel Nyamai, Dorothy Okello, Wilson Oywer, Lordin Wanjala

**Affiliations:** 1 Health Services Unit, Kenya Medical Research Institute (KEMRI)–Wellcome Trust Research Programme, Nairobi; 2 Medical Research Council Tropical Epidemiology Group, Department of Infectious Disease Epidemiology; 3 Department of Medical Statistics, London School of Hygiene and Tropical Medicine, United Kingdom; 4 Department of Paediatrics and Child Health, University of Nairobi, Kenya; 5 Nuffield Department of Medicine, University of Oxford, United Kingdom

**Keywords:** childhood pneumonia, sub-Saharan Africa, amoxicillin, treatment failure, World Health Organization

## Abstract

***Background.*** There are concerns that the evidence from studies showing noninferiority of oral amoxicillin to benzyl penicillin for severe pneumonia may not be generalizable to high-mortality settings.

***Methods.*** An open-label, multicenter, randomized controlled noninferiority trial was conducted at 6 Kenyan hospitals. Eligible children aged 2–59 months were randomized to receive amoxicillin or benzyl penicillin and followed up for the primary outcome of treatment failure at 48 hours. A noninferiority margin of risk difference between amoxicillin and benzyl penicillin groups was prespecified at 7%.

***Results.*** We recruited 527 children, including 302 (57.3%) with comorbidity. Treatment failure was observed in 20 of 260 (7.7%) and 21 of 261 (8.0%) of patients in the amoxicillin and benzyl penicillin arms, respectively (risk difference, −0.3% [95% confidence interval, −5.0% to 4.3%]) in per-protocol analyses. These findings were supported by the results of intention-to-treat analyses. Treatment failure by day 5 postenrollment was 11.4% and 11.0% and rising to 13.5% and 16.8% by day 14 in the amoxicillin vs benzyl penicillin groups, respectively. The most frequent cause of cumulative treatment failure at day 14 was clinical deterioration within 48 hours of enrollment (33/59 [55.9%]). Four patients died (overall mortality 0.8%) during the study, 3 of whom were allocated to the benzyl penicillin group. The presence of wheeze was independently associated with less frequent treatment failure.

***Conclusions.*** Our findings confirm noninferiority of amoxicillin to benzyl penicillin, provide estimates of risk of treatment failure in Kenya, and offer important additional evidence for policy making in sub-Saharan Africa.

***Clinical Trial Registration.*** NCT01399723.

## Abstract (precis)

Evidence demonstrating noninferiority of oral amoxicillin vs benzyl penicillin for severe childhood pneumonia is largely drawn from Asian populations where mortality is low. This study confirms noninferiority and is expected to inform policy on treatment of pneumonia in sub-Saharan Africa.

**(See the Editorial Commentary by Qazi, Fox, and Thea on pages 1225–7.)**

Almost half of all deaths due to childhood pneumonia occur in sub-Saharan Africa [1]. World Health Organization (WHO) recommendations for case management of pneumonia in children aged 2–59 months have been credited with contributing to substantial reductions in mortality [2]. For >2 decades, these guidelines have recommended classification into 4 classes of severity based on clinical signs at initial presentation, with inpatient management reserved for the severe categories (Figure 1) [3].

**Figure 1.**
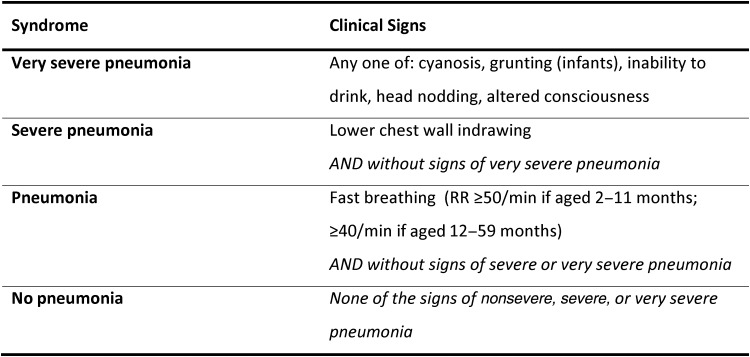
Kenyan Ministry of Health classification of pneumonia for children aged 2–59 months with cough and/or difficulty breathing (without stridor, severe malnutrition, or signs of meningitis). Abbreviation: RR, respiratory rate.

Recently, however, WHO undertook a major revision of these guidelines. Children with lower chest wall indrawing are now to be treated with outpatient oral amoxicillin, replacing inpatient benzyl penicillin [4]. Supporting evidence came from large multicenter studies including >3000 children, predominantly from Asian countries [5, 6]. The WHO panel utilizing the Grading of Recommendations Assessment Development and Evaluation process [7] was moderately confident in these effect estimates and provided a strong recommendation in favor of this policy shift [8].

The same evidence was reviewed and discussed in Kenya in 2010 as part of a national guideline development exercise. In this context-specific decision-making process, the supporting evidence was also graded as of moderate quality, with downgrading for indirectness given the small number of African children involved in the trials. A panel of >70 individuals from clinical, policy, and academic backgrounds who participated in this national process had concerns over the generalizability of the results drawn from predominantly Asian study subjects to populations in sub-Saharan Africa in whom mortality may be higher [9]. On this background, the Kenyan group declined to adopt outpatient oral amoxicillin to replace injectable benzyl penicillin [10].

Prompted by this uncertainty, we sought to compare the 2 antibiotics in a population of Kenyan children. Of specific interest, this trial provides the first data on clinical outcomes of children treated for severe pneumonia in an African setting where comorbid conditions such as malaria, diarrhea, and malnutrition are common and was conducted after introduction of the routine childhood *Haemophilus influenzae* type b (Hib) and pneumococcal vaccines in Kenya.

## METHODS

### Participants

We conducted an open-label, pragmatic, randomized controlled noninferiority trial. Children aged 2–59 months with severe pneumonia as defined in the 2005 WHO guidelines were recruited from 6 public hospitals across Kenya. Three of the sites are located in the central region of Kenya at elevations ranging from 1350 m to 1700 m above sea level. The other 3 hospitals are located in western Kenya around the Lake Victoria basin where malaria is endemic (1100 m–1300 m above sea level). All 6 facilities offer both primary care and referral services, with pediatric inpatient departments reporting 2500–4500 annual pediatric admissions. Patient screening and enrollment was conducted by trained study clinicians under supervision of the hospital pediatricians who were also the site principal investigators.

### Eligibility Criteria

To mimic scenarios encountered in actual practice, we enrolled children with common comorbidities including malaria, diarrhea, wheeze, and a single convulsion in the presence of fever. As some form of treatment is common prior to consulting a health professional, children with a recent history of treatment with unknown oral antibiotics or those who had received <48 hours of amoxicillin were enrolled. Although enrolling children with comorbidities or oral pretreatment would potentially bias the results of the trial toward equivalence, we considered the value of gaining data on the population who would actually become the subject of future guidelines of overriding interest. To minimize this bias, we ensured prompt and adequate management of comorbidities. Children with primary diagnoses that would ordinarily preclude treatment with benzyl penicillin monotherapy were excluded (Supplementary Figure 1).

All children were assessed for the presence of wheeze. Where present, up to 3 doses of inhaled salbutamol were administered as bronchodilator therapy, each separated by 15-minute intervals, at the end of which they were reassessed. Those in whom signs of severe pneumonia subsided exited the study with inhaled bronchodilators and corticosteroids, in line with recommended practice [3].

### Study Oversight

The Kenya Medical Research Institute (KEMRI) National Ethical Review Committee (ERC), Kenya National Pharmacy and Poisons Board, and University of Oxford Tropical Research Committee approved the trial protocol. Written informed consent was sought from accompanying legal guardians in a locally appropriate language prior to enrollment. All serious adverse events were reported to the KEMRI ERC and the data and safety monitoring board (DSMB). One planned interim analysis was conducted upon recruitment of approximately half of the total sample size. Using the Haybittle–Peto adjustment for multiple testing, recruitment was to be halted if the *P* value from a test of difference between the 2 groups was ≤.001 [11]. Upon reviewing the report, the DSMB expressed satisfaction with the findings and recommended continuation of recruitment with no amendments to the protocol. Independent monitoring was provided by the Clinical Trials Facility of the KEMRI–Wellcome Trust Research Programme, Kilifi, Kenya. The trial is registered with ClinicalTrials.gov (NCT01399723).

### Interventions

**Figure 2.**
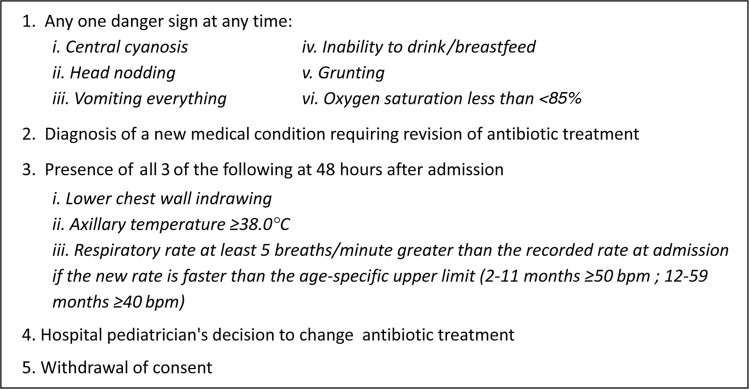
Criteria for treatment failure. Abbreviation: bpm, breaths per minute.

Eligible children were randomized to oral amoxicillin at the WHO-recommended dose of 40–45 mg/kg twice daily or intravenous/intramuscular benzyl penicillin at 50 000 IU/kg 4 times daily for a minimum of 48 hours. Additional supportive care and laboratory and radiological investigations were performed as determined by the clinical team managing the ward patients. The clinical team was trained and advised to strictly adhere to the study protocols and ensure that any change of treatment was based on the study definitions of treatment failure (Figure 2).

The study clinician conducted daily clinical reviews in consultation with the hospital pediatrician. Study participants were monitored for signs of clinical deterioration, which resulted in prompt revision of treatment if detected. Follow-up data on inpatient treatment failure and mortality continued until discharge from hospital or day 5 postenrollment. At hospital discharge, children in both study arms received adequate oral amoxicillin to complete a total of 5 days of antibiotic treatment. Guardians were counseled on correct administration of and adherence to treatment prior to discharge. Mobile phone contacts collected from the children's parents or legal guardians were used to facilitate follow-up and capture information on any deaths and readmissions both within and outside of the recruiting hospitals through a telephone interview 2 weeks after enrollment. Caregivers of patients who we knew at discharge were not contactable by telephone were requested to report to the hospital 2 weeks after enrollment for a direct interview. Participants were compensated for transport costs incurred to and from hospital for this visit.

### Study Drugs

Trial lots of amoxicillin and benzyl penicillin were procured directly from the manufacturer with accompanying batch release certificates. Quality of both study treatments was verified at a local WHO-accredited laboratory.

### Outcomes

The primary outcome was treatment failure determined by the study site clinician in discussion with the site principal investigator at 48 hours (2 full days after enrollment). Treatment failure was defined a priori as the development of any 1 of 5 prespecified criteria (Figure 2). The secondary outcomes were cumulative treatment failure 5 days after enrollment or upon hospital discharge (whichever occurred sooner) and late treatment failure at day 14, defined as death or prolonged hospitalization, or hospital readmission, or ongoing treatment with outpatient antibiotics as determined through telephone or direct interview.

### Statistical Analysis

Data on baseline characteristics were summarized by study arm. Noninferiority between amoxicillin and benzyl penicillin was defined a priori as a risk difference of treatment failure and associated upper bound of the 95% confidence interval (CI) of <7%. This definition is comparable to those of previous studies on childhood pneumonia [5, 6, 12–15]. The initial sample size estimate of 576 children (288 per group) would provide 80% power to detect noninferiority of amoxicillin against benzyl penicillin within a margin of 7% at a 1-sided level of significance of 0.025, assuming a prevalence of treatment failure of 10% at 48 hours derived from a preintervention pilot phase of the study. We undertook both intention-to-treat and per-protocol analyses for the primary outcome.

We also explored independent risk factors for treatment failure at 48 hours using a predictive logistic regression model. Age, sex, and treatment group were included as a priori covariates. Univariate associations were tested for other potential predictors of treatment failure. An inclusion cutoff of *P* < .1 was applied to select covariates for inclusion in the final model. All analyses were conducted using Stata version 12.1 (StataCorp, College Station, Texas).

### Randomization

Computer-generated random sequences were created in blocks of randomly varying sizes of 6–10, stratified by study site at the KEMRI–Wellcome Trust Research Programme by an individual independent of the investigators. Treatment allocations were stored in sealed opaque envelopes distributed to the study sites in complete blocks. Envelopes were issued to recruited participants in order of enrollment. Due to the nature of the intervention (injectable vs orally administered treatments), blinding was not achieved. However, envelopes containing the assigned treatment were only opened by the recruiting clinician after a potential study patient was determined to have fully satisfied eligibility criteria ensuring allocation concealment. A screening log was maintained to show corresponding accountability for all opened allocation envelopes.

## RESULTS

The trial was preceded by a pilot phase during which we recruited 208 children, in whom we observed a failure rate of 10.1% (95% CI, 6.4–15.0) and 2 deaths. Characteristics of the children recruited in the pilot phase were similar to those in the trial.

**Figure 3.**
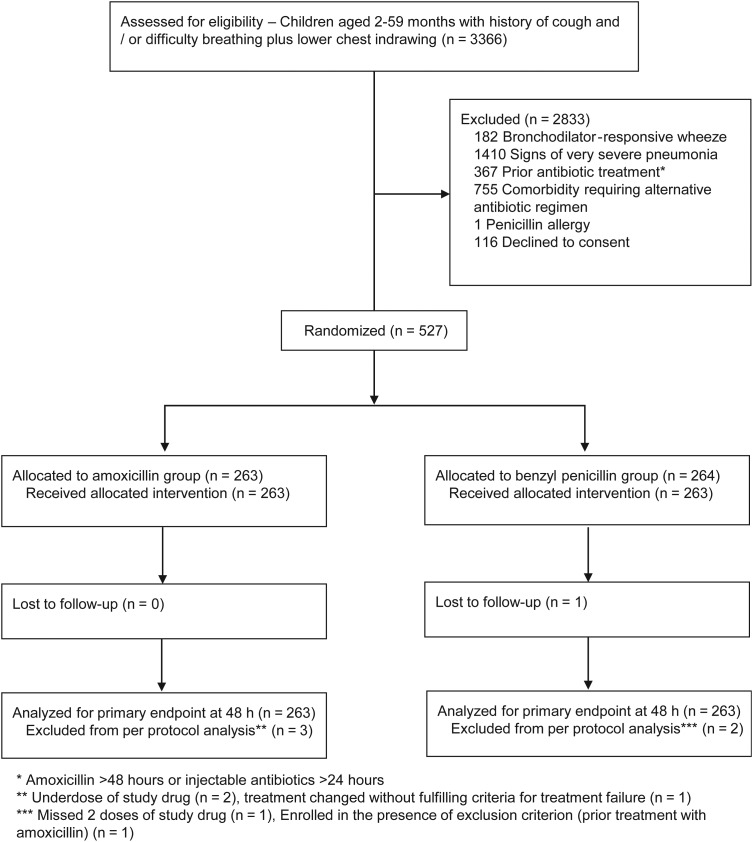
Screening allocation and follow-up of study participants.

During the intervention phase of the study, 527 were recruited from 12 September 2011 to 15 August 2013. A total of 263 children were randomized to receive amoxicillin whereas 264 received benzyl penicillin. One patient in the benzyl penicillin group was lost to follow-up before assessment for the primary outcome at 48 hours (Figure 3). Recruitment was adversely affected by several unpredictable nationwide health worker strikes during the study period. At one hospital, recruitment stopped after the site clinician resigned from his position (8 months before the end of the recruitment). Faced with uncertainty regarding the expected end of the trial and a limited budget to prolong recruitment indefinitely, we decided to base the end of the trial on a fixed date rather than achieving the target sample size. No data analysis preceded study termination. Thus, we concluded recruitment with a sample size of 527 (49 patients below the original target).

Protocol violations were observed in 5 patients. Two patients received inadequate doses of study drug, 1 patient missed 2 doses of study drug, treatment for 1 patient was changed in the absence of any criteria for treatment failure, and 1 patient was enrolled despite having been treated with oral amoxicillin for >48 hours prior to admission. Data from these patients were excluded from per-protocol analyses.

Comorbidities including malaria (confirmed through microscopy), acute diarrheal illness, wheeze not responsive to initial therapy, anemia, and moderate acute malnutrition were present in 302 of 527 (57.3%) of study patients, rates of comorbidity consistent with previous reports in similar populations [16, 17]. Baseline characteristics for recruited children were comparable between the treatment groups (Table 1).

**Table 1.** Baseline Characteristics of Recruited Children

Patient Characteristic	Penicillin	Amoxicillin
Age, mo, median (IQR)	13 (7–24)	14 (7–25)
Female sex	106/264 (40.2)	120/263 (45.6)
History of cough	263/264 (99.6)	262/263 (99.6)
History of fever	240/264 (90.9)	236/263 (89.7)
History of diarrhea	62/264 (23.5)	56/263 (21.3)
Recent antibiotic treatment	103/236 (43.6)	101/238 (42.4)
Received pneumococcal and Hib vaccines, ≥1 dose	184/245 (75.1)	183/245 (74.7)
Temperature, °C, median (IQR)	37.8 (37.3–38.5)	38.1 (37.5–38.8)
Oxygen saturation, %, median (IQR)	94 (92–96)	95 (93–97)
WHZ < −2 SD below median reference value	24/264 (9.0)	15/263 (5.7)
Any comorbidity	150/264 (56.8)	152/263 (57.8)
Wheeze	92/264 (34.9)	95/263 (36.1)
Slide-positive malaria	22/264 (8.3)	34/263 (12.9)
Pallor	20/264 (7.6)	19/263 (7.2)
Dehydration	20/264 (7.5)	11/263 (4.2)
Study site		
Bungoma District Hospital	42/264 (15.9)	41/263 (15.6)
Embu Provincial General Hospital	62/264 (23.1)	60/263 (22.8)
Kerugoya District Hospital	60/264 (22.7)	59/263 (22.4)
Kisumu District Hospital	37/264 (14.0)	36/263 (13.7)
Mbagathi District Hospital	39/264 (14.8)	41/263 (15.6)
New Nyanza Provincial General Hospital	25/264 (9.5)	26/263 (9.9)

Data are presented as proportion (%) unless otherwise indicated.

Abbreviations: Hib, *Haemophilus influenzae* type b; IQR, interquartile range; SD, standard deviation; WHZ, weight-for-height *z* score.

Of 11 patients with oxygen saturation recordings of 85%–90%, treatment failure was observed in 1 patient (9.1%). This finding was not statistically different from the risk of treatment failure among children with oxygen saturations ≥90% (*P* = .87).

### Main Outcomes

**Figure 4.**
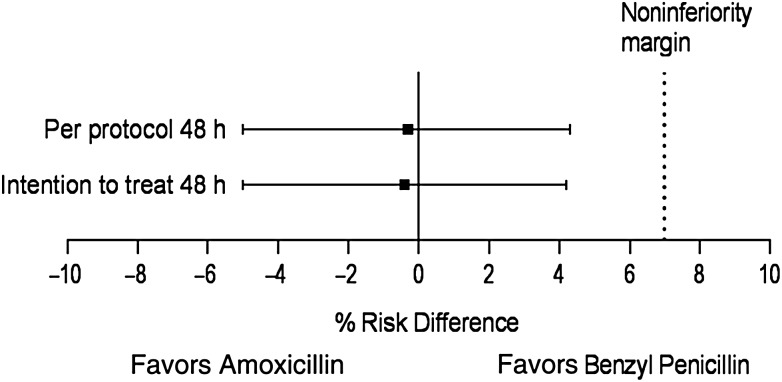
Intention-to-treat and per-protocol analyses for treatment failure at 48 hours.

The risk of treatment failure was 7.7% in the amoxicillin arm and 8.0% in the benzyl penicillin arm at 48 hours in per-protocol analyses (risk difference, −0.3% [95% CI, −5.0% to 4.3%]), indicating noninferiority within the prespecified margin of 7% at 48 hours (Figure 4). Similar results were obtained in intention-to-treat analyses. The risk difference of treatment failure between the 2 treatment arms at all follow-up time-points was <7%: −0.3% at 48 hours, 0.4% at day 5, and 3.3% at day 14 (Table 2).

**Table 2.** Cumulative Treatment Failure at 48 Hours, Day 5, and Day 14

Treatment Group	48 h, PP, Proportion (%)	48 h, ITT, Proportion (%)	Day 5, ITT, Proportion (%)	Day 14, ITT, Proportion (%)
Amoxicillin	20/260 (7.7)	20/263 (7.6)	30/263 (11.4)	33/244 (13.5)
Penicillin	21/261 (8.0)	21/263 (8.0)	29/263 (11.0)	42/250 (16.8)
Risk difference, % (95% CI)	−0.3 (−5.0 to 4.3)	−0.4 (−5.0 to 4.2)	0.4 (−5.0 to 5.8)	−3.3 (−10.0 to 3.0)

Abbreviations: CI, confidence interval; ITT, intention to treat; PP, per protocol.

### Risk Factor Analysis

In univariate analyses, moderate acute malnutrition (weight-for-height *z* score ≥ −3 SD and < −2 SD vs ≥ −2 SD) and history of diarrhea were associated with increased odds of treatment failure (odds ratio [OR], 2.91 [95% CI, 1.19–7.13]; *P* = .01 and OR, 2.4 [95% CI, 1.23–4.67]; *P* = .01, respectively). The presence of wheeze was associated with reduced odds of treatment failure (OR, 0.18 [95% CI, .06–.51]; *P* = .001). Treatment failure was weakly associated with palmar pallor (OR, 2.35 [95% CI, .92–6.0]; *P* = .07) and increasing length of illness in days (OR, 1.13 [95% CI, .98–1.30]; *P* = .08). Immunization status (pneumococcal or Hib vaccines), laboratory diagnosis of malaria, and recent antibiotic treatment were not associated with treatment failure.

A multivariable predictive model was used to determine independent correlates of treatment failure adjusting for treatment group, age, sex, length of illness, history of diarrhea, temperature, wheeze at the time of recruitment, moderate acute malnutrition, and a diagnosis of anemia. In the final model, only presence of wheeze was found to be associated with reduced risk of treatment failure (OR, 0.21 [95% CI, .07–.63]; *P* = .005).

### Causes of Treatment Failure

Approximately half (33/59) of the children who progressed, cumulatively, to treatment failure were reported to have deteriorated, developing at least 1 of the signs of very severe pneumonia; 28 of these deteriorated within the first 48 hours of admission. In contrast, change of treatment by a clinician in the absence of any of the trial-specified criteria for treatment failure was observed more frequently as a late cause (>48 hours after admission) of treatment failure. Two patients (1 from each study group) withdrew consent during the study (Table 3).

**Table 3.** Day of Occurrence and Reasons for Treatment Failure

Reason for Treatment Failure	Day Postenrollment					Total		
	1	2	3	4	5	Amoxicillin	Benzyl Penicillin	All
Signs of very severe pneumonia	17	11	4 (1^a^)	1	0	18	15	33
Persisting distress	0	4	0	0	0	2	2	4
Clinician's decision	1	2	3	3	2	7	4	11
Change of diagnosis	5	0	3	1	0	1	8	9
Withdrawal of consent	1	0	1	0	0	1	1	2
Total	24	17	11	5	2	29	30	59

^a^ Death following treatment failure.

### Late Treatment Failure and Mortality

**Figure 5.**
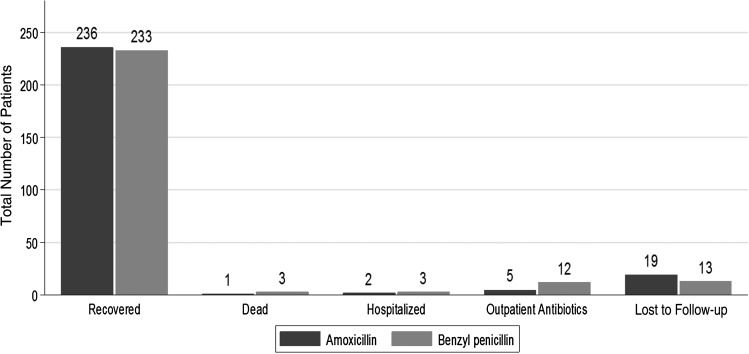
Outcome of recruited patients on day 14 postenrollment.

Late treatment failure, assessed 14 days after enrollment, was more frequent, although not statistically significantly, in the benzyl penicillin group (Table 2). However, the proportion of children lost to follow-up by day 14 was slightly greater in the amoxicillin group at 7.2% (19/263) vs 4.3% (13/264) (Figure 5).

Four study participants died (0.8%): 3 in the benzyl penicillin group and 1 in the amoxicillin group. None of the deaths occurred in the first 48 hours of treatment. One death occurred after hospital discharge, 12 days after recruitment. Cause of death was ascribed to complications of acute diarrhea (n = 1), congestive heart failure (n = 1), complications of chronic renal failure (n = 1) unrecognized at enrollment, and possible intoxication related to ingestion of traditional herbal medicines (n = 1). All deaths were reported to the DSMB and the KEMRI ERC, who in all instances concluded that none of the deaths was related to study drugs or procedures.

## DISCUSSION

The results of this trial are consistent with findings of other large multicenter studies [5, 6, 18] that informed a recent evidence-driven review of the treatment guidelines for severe pneumonia by WHO, recommending outpatient oral amoxicillin [8]. We have included our new data in a random-effects meta-analysis with the 2 previous comparable trials [5, 6] (Supplementary Figure 2). The pooled risk difference for treatment failure comparing amoxicillin vs benzyl penicillin is −0.6% (95% CI, −2.4% to 1.2%; *I*^2^ = 0%, *P* = .77).

The risk of treatment failure in the trial was lower than the estimate obtained from the pilot from which the initial sample size calculation was derived. This implies that the number of patients recruited, although lower than the original target, may have been adequate to achieve the study objectives. However, the overall risk of treatment failure of almost 10% still represents a substantial amount of suffering in both treatment groups. Thus, the need remains for research to examine complementary interventions to further reduce poor outcomes among children with severe forms of pneumonia such as optimal feeding and fluid regimens.

We excluded children with severe acute malnutrition and alternative or additional severe illnesses according to Kenyan case management guidelines [3] (which are broadly comparable with WHO guidelines [4]). Exploratory analyses for factors associated with treatment failure were thus limited to milder levels of risk. This may explain why factors that have traditionally been associated with poor clinical outcomes were not found to be predictive of treatment failure in our study. Many Kenyan hospitals are unable to perform routine pulse oximetry [19]. In line with the pragmatic design of the study, we therefore included children without signs of very severe pneumonia but with a “spot” oxygen saturation on admission examination as low as 85% with no evidence of resultant harm. The presence of wheeze was found to have a protective association with treatment failure, a finding that is consistent with work previously conducted in South Africa to develop a clinical predictive score for mortality in children with lower respiratory tract infection [20].

Randomized controlled trials are frequently criticized for their lack of external validity [21] due to strict eligibility criteria and differences between trial protocols and routine care. To ensure that the trial was conducted under conditions representative of typical settings in district-level hospitals across Africa with large patient volumes and diverse clinical presentations, we adopted a pragmatic design. Whereas 1 study clinician per site reviewed trial patients, daily further clinical and nursing management was undertaken by hospital staff with access to routinely available resources only and with care supervised by the hospital pediatrician. The ability to provide policy makers data on outcomes of treatment in typical settings where children often have comorbid conditions as well as information on the comparative effectiveness of treatments was thought to be critical at the outset given the prior rejection of suggested policy changes despite moderate quality trial data [10]. The results of this trial therefore provide much-needed evidence for pneumonia guideline development discussions in Kenya and the sub-Saharan Africa region. Overall mortality was comparable to that reported in the APPIS trial, a multicountry trial that included children with human immunodeficiency virus, in which 0.7% of participants died. Mortality in other cohorts of children with severe pneumonia is low, ranging from 0% to 0.2% [5, 18, 22, 23]. More than half of those who failed treatment were observed to have developed signs of very severe pneumonia (6.2% of enrolled children) requiring broad-spectrum parenteral antibiotics and, in many cases, oxygen. The challenge of determining the contribution of these more aggressive treatment options to the patients' ultimate clinical outcome if children are treated from the outset as outpatients, as now recommended by WHO, will warrant consideration as countries develop contextually appropriate policies.

Negative views regarding the efficacy of oral vs injectable medication may have influenced decisions to declare treatment failure among children treated with amoxicillin more readily than in those receiving benzyl penicillin in this open-label study. However, misclassification of this nature is not expected to have had an effect on the results of the study, as it would bias the results in favor of the standard treatment, benzyl penicillin.

## CONCLUSIONS

Oral treatment of severe pneumonia offers several potential benefits including the alternative of outpatient treatment, reduced demand for nursing care, elimination of the risks associated with injectable medications, and a potential reduction in the overall cost of treatment. The findings of this study are consistent with those of previous trials suggesting similar outcomes for severe pneumonia treated with benzyl penicillin and oral amoxicillin. The pragmatic nature of the study and associated data on the frequency and nature of treatment failure will provide important new evidence to support discussions on appropriate guidance for Kenya and, potentially, other countries with similar patient populations and health systems.

## Supplementary Data

Supplementary materials are available at *Clinical Infectious Diseases* online (http://cid.oxfordjournals.org). Supplementary materials consist of data provided by the author that are published to benefit the reader. The posted materials are not copyedited. The contents of all supplementary data are the sole responsibility of the authors. Questions or messages regarding errors should be addressed to the author.

Supplementary Data
