# Supplementary material for: Oral Amoxicillin Versus Benzyl Penicillin for Severe Pneumonia Among Kenyan Children: A Pragmatic Randomized Controlled Noninferiority Trial
Source: Clin Infect Dis. 2014 Dec 30;60(8):1216–24. doi: 10.1093/cid/ciu1166 (PMC4370168; doi:10.1093/cid/ciu1166)
Supplement: Supplementary Data [file supp_60_8_1216__index.html]

Oral Amoxicillin Versus Benzyl Penicillin for Severe Pneumonia Among Kenyan Children: A Pragmatic Randomized Controlled Noninferiority Trial — Oral Amoxicillin Versus Benzyl Penicillin for Severe Pneumonia Among Kenyan Children: A Pragmatic Randomized Controlled Noninferiority Trial — Supplementary Data 

# Oral Amoxicillin Versus Benzyl Penicillin for Severe Pneumonia Among Kenyan Children: A Pragmatic Randomized Controlled Noninferiority Trial

## Supplementary Data

Supplementary Data

**Files in this Data Supplement:**

- Supplementary Figure 1 - pptx file
- Supplementary Figure 2 - tif file
