# Supplementary figures and images for: Oral Amoxicillin Versus Benzyl Penicillin for Severe Pneumonia Among Kenyan Children: A Pragmatic Randomized Controlled Noninferiority Trial
Source: Clin Infect Dis. 2014 Dec 30;60(8):1216–24. doi: 10.1093/cid/ciu1166 (PMC4370168; doi:10.1093/cid/ciu1166)

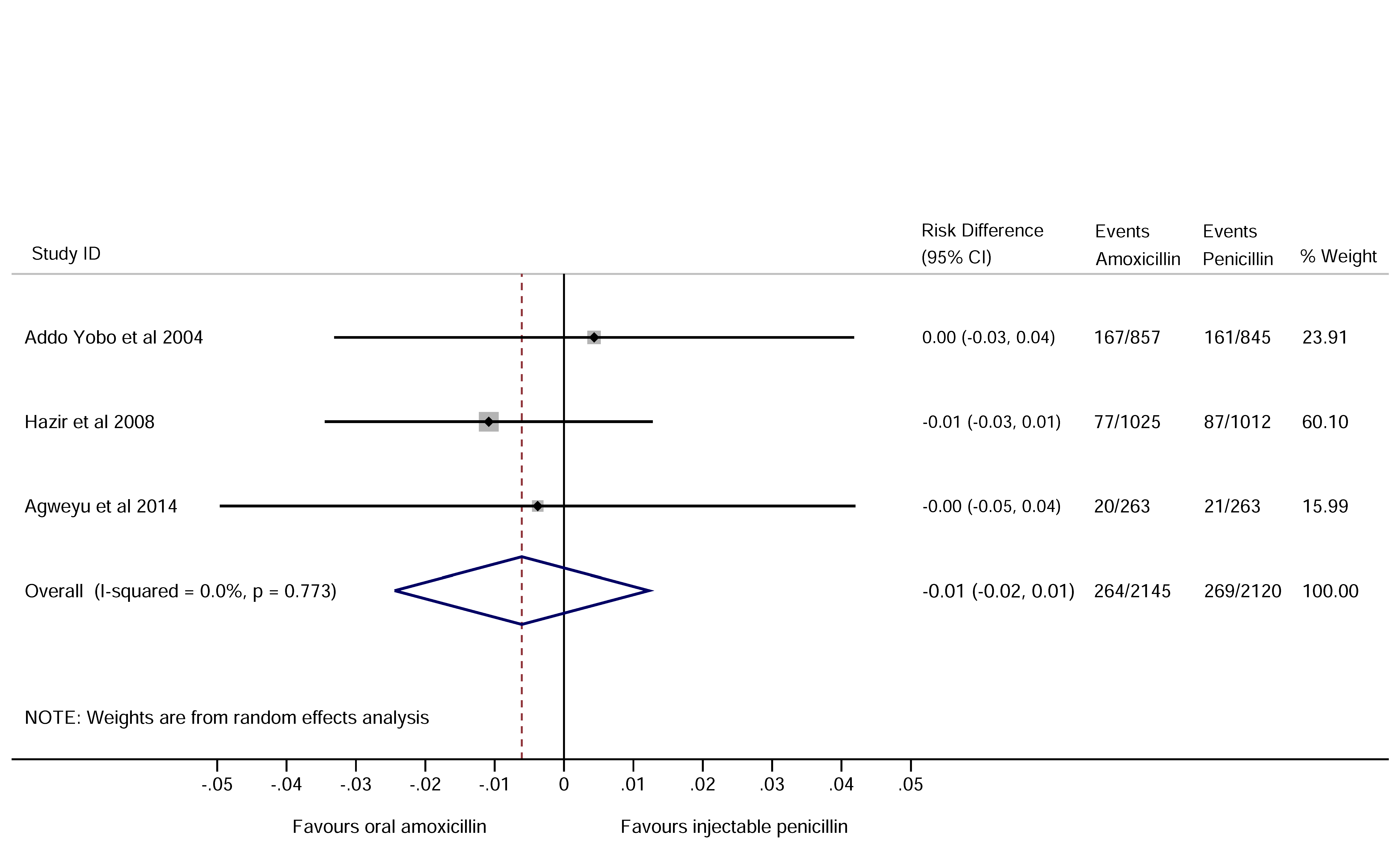

Supplement: Supplementary Data [file supp_ciu1166_ciu116supp_fig2.tif]
