# Supplementary material for: Oral Amoxicillin Versus Benzyl Penicillin for Severe Pneumonia Among Kenyan Children: A Pragmatic Randomized Controlled Noninferiority Trial
Source: Clin Infect Dis. 2014 Dec 30;60(8):1216–24. doi: 10.1093/cid/ciu1166 (PMC4370168; doi:10.1093/cid/ciu1166)
Supplement: Supplementary Data [file supp_ciu1166_ciu1166supp_fig1.pptx]

## Slide 1
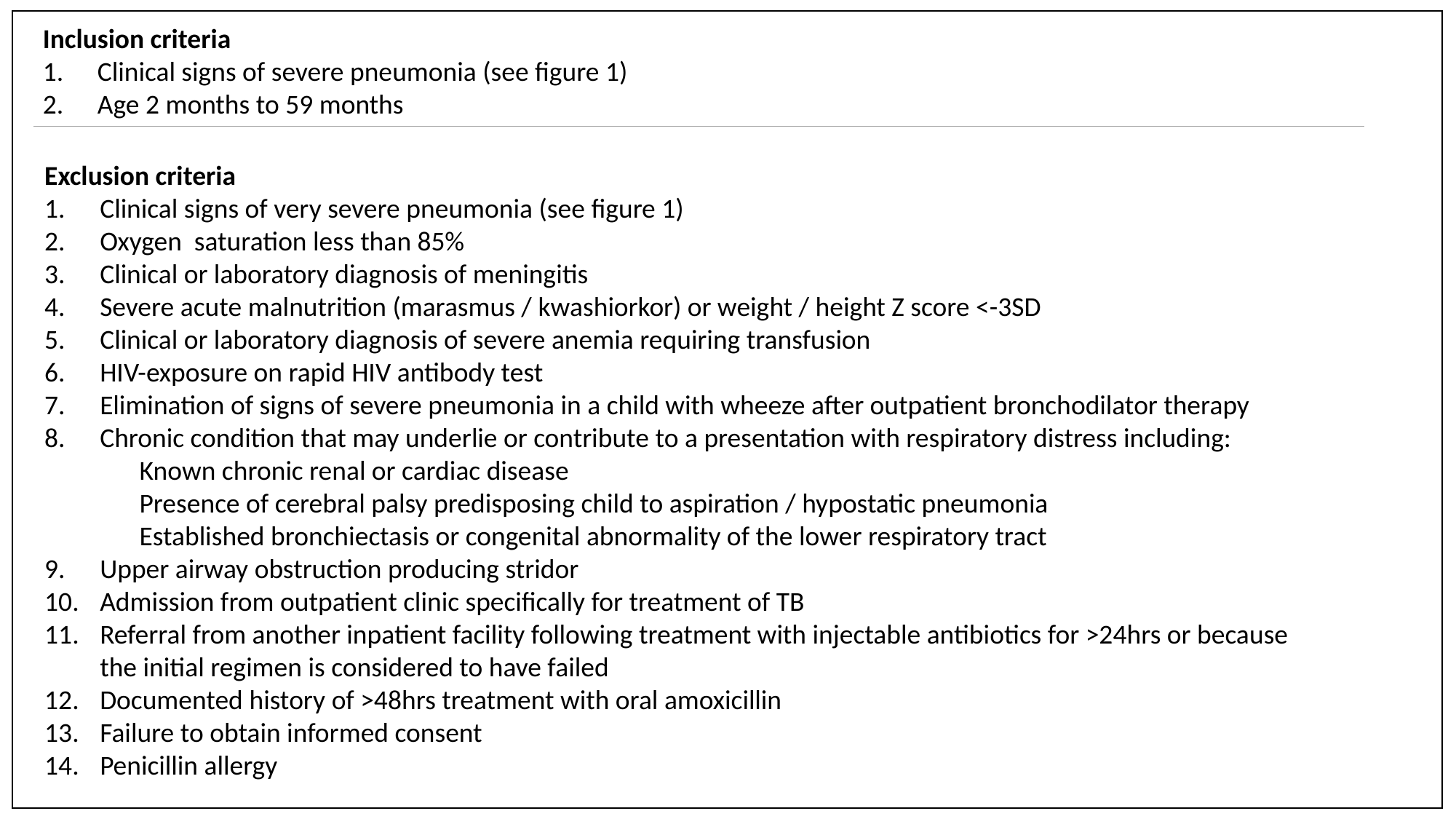

Inclusion criteria
Clinical signs of severe pneumonia (see figure 1)
Age 2 months to 59 months
Exclusion criteria
Clinical signs of very severe pneumonia (see figure 1)
Oxygen saturation less than 85%
Clinical or laboratory diagnosis of meningitis
Severe acute malnutrition (marasmus / kwashiorkor) or weight / height Z score <-3SD
Clinical or laboratory diagnosis of severe anemia requiring transfusion
HIV-exposure on rapid HIV antibody test
Elimination of signs of severe pneumonia in a child with wheeze after outpatient bronchodilator therapy
Chronic condition that may underlie or contribute to a presentation with respiratory distress including:
Known chronic renal or cardiac disease
Presence of cerebral palsy predisposing child to aspiration / hypostatic pneumonia
Established bronchiectasis or congenital abnormality of the lower respiratory tract
Upper airway obstruction producing stridor
Admission from outpatient clinic specifically for treatment of TB
Referral from another inpatient facility following treatment with injectable antibiotics for >24hrs or because the initial regimen is considered to have failed
Documented history of >48hrs treatment with oral amoxicillin
Failure to obtain informed consent
Penicillin allergy
